# A pilot study of 17 wrist-cutting suicide injuries in single institution: perspectives from a hand surgeon

**DOI:** 10.1186/s12873-021-00432-4

**Published:** 2021-03-31

**Authors:** Jong-Ho Kim, Hyokyung Yoo, Seokchan Eun

**Affiliations:** Department of Plastic and Reconstructive Surgery, Seoul National University College of Medicine, Seoul National University Bundang Hospital, 82 Gumi-ro 173beon-gil, Bundang-gu, Seongnam, 463-707 South Korea

**Keywords:** Wrist cutting, Mental disorders, Suicide, Wrist injury, Hand surgery

## Abstract

**Background:**

Self-cutting is a special type of emergency in hand surgery. Despite its low mortality rate, it is clinically significant because there is a possibility of permanent disability and repeated suicide attempts are likely to occur. Therefore, we aim to understand the characteristics of self-inflicted wrist injuries and share the perspectives from a hand surgeon in order to inform those who face these patients primarily in the emergency room.

**Methods:**

We reviewed 17 patients with self-inflicted wrist injuries who were referred to the Department of Hand surgery from the Emergency Medicine Department from 2013 to 2017. We investigated the differences in demographic features (age, gender, psychological diagnosis, alcohol consumption, prior suicide attempts) and clinical features (injury side, injury pattern, anatomical structures, distance from wrist crease).

**Results:**

Among the patients, 4 were female and 13 were male. 70.6% of patients (12/17) had injuries on the left wrist and 94.1% of patients (16/17) had injuries on the flexor side. The average distance from the wrist crease to the injured site was 3.43 cm and 90.5% (19/21) of total injuries had an average distance of was less than 5 cm. The most frequently injured structures were palmaris longus tendon (58.5%, 10/17). 52.9% (9/17) of patients, among which 6 of the 8 patients with deep injuries and 3 of the 9 patients with superficial injuries, had a history of a psychiatric disorder.

**Conclusions:**

We conclude that a male with a previously diagnosed psychiatric disorder has a higher chance of inflicting a deeper injury. In addition, self-cutting injuries are highly predictable because most of these injuries occur on the flexor side of the left wrist and are limited to a distance of 5 cm from the wrist crease. In terms of the implements used in self-inflicted injuries, we can predict the type of damage to some degree depending on the type of implement used. In view of these characteristics, more appropriate evaluation can be implemented in the emergency room and those who deal with these patients primarily can cope more effectively for better long-term results.

## Background

Suicide is a major public health concern and the increase of suicide cases is a serious social problem. It is the 10th leading cause of death worldwide and about one million people died from suicide every year [1]. In Korea, suicide has been the fourth leading cause of death and the most common cause in adolescents [2]. The most common causes of the patients who attempted suicide were self-poisoning or self-wrist cutting [3]. On average, there are 20–25 suicide attempts for every completed suicide, frequently by self-inflicted wrist cutting [4]. In the United States, the number of patients who attempted suicide and self-inflicted injury increased significantly over the past few decades [5, 6]. Demographically, wrist-cutting suicide injuries were more common for under the age of 20 and females [5]. Self-cutting injuries have a low mortality rate, which means that most of suicide attempts end in survival. In surviving patients, this is a clinically significant problem because of the risk of permanent disabilities and the repetition of suicide attempts [7]. Self-inflicted wrist cutting injuries may vary from simple skin lacerations to deep wrist injuries. Consequently, this has a strong effect on the anatomical structures such as arteries, tendons, and nerves, which can lead to motor and sensory dysfunction. Such patients have impaired ability not only to maintain work, hobbies or social activities but also to perform basic activities in daily life. Thus, wrist cutting injuries should be managed in terms of both psychological intervention and wound treatment [8]. For psychiatric diagnosis and treatment, all patients who are admitted to the emergency department for attempted suicide should be assessed by a psychiatrist [9]. In addition, in order to prevent any functional impairment, an initial appropriate evaluation and proper referral are of pivotal importance. Thus, the objective of this study was to investigate the characteristics of self-inflicted wrist injuries in a single institution and share the perspectives from a hand surgeon so that those who deal with these patients primarily in the emergency room can manage these injuries more appropriately.

## Methods

We investigated all self-inflicted wrist injury patients who were referred to the Department of Hand Surgery from the Emergency Medicine Department in Seoul National University Bundang Hospital from 2013 to 2017. This study was conducted as a pilot study before a prospective study in the same institution had been initiated from 2017. The patients who had skin only injuries were excluded because primary closure was performed at the Emergency Medicine Department. We derived all information from the patients’ medical records, examinations, and interviews including psychiatric consultation at the time of examination. Demographic data (age, gender, alcohol intake, psychological state) and clinical features (injury side, injury pattern, anatomical structures involved, distance from wrist crease) were analyzed. A psychological evaluation of all the patients was performed following attempted suicide by a psychiatrist from the Department of Psychiatry within the same institution. Patients were initially assessed in the Emergency Medicine Department and surgical treatment and postoperative wound care were performed by the Hand Surgery Department of Plastic and Reconstructive Surgery Department in the same institution. The outpatient follow-up period was at least three months and postoperative long-term disability was evaluated. The long-term motor function was assessed by range of motion, opposition of the thumb, intrinsic function tests. Two-point discrimination test was performed in order to evaluate sensory function.

## Results

A total of 17 patients who attempted suicide by cutting their wrists were included in our study. The ages ranged from 19 to 72 years (mean age, 38 yr). Among the patients, four were females and 13 were males. The left wrist was involved in 70.6% (12/17) of cases, the right wrist in 11.8% (2/17) and both wrists were involved in the remaining 17.6% (3/17) of cases. In 94.1% of the cases (16/17) the injuries involved the flexor side of the forearm: among these patients, eight (patient no. 1–8) had ‘deep’ injuries involving deep flexor tendons, the median nerve, the ulnar nerve, the radial artery or the ulnar artery while the remaining patients had ‘superficial’ injuries that extended at most to the subcutaneous tissue and superficial tendon groups (palmaris longus, flexor carpi radialis and flexor carpi ulnaris tendon). Only in one case the injury occurred on the radial side of the forearm with involvement of the abductor pollicis longus and the extensor pollicis longus tendons. 61.5% (8/13) of male patients had injuries involving deep anatomical structures, whereas there was no deep wrist injury in female patients. The average distance from the wrist crease to the injured site was 3.43 cm, and 90.5% (19/21) of total injuries had a distance of less than 5 cm. The most frequently injured structures was the palmaris longus tendon (58.5%, 10/17), followed by the flexor carpi radialis (35.3%, 6/17). The most frequently injured nerve was the median nerve (23.5%, 4/17). The ulnar neuro-vascular bundle and the radial artery were involved only once each. Injuries of important anatomical structures are summarized in Table 1. Knife was the most common tool for suicide attempts, followed by glass (Table 2). Alcohol intake prior to suicide attempts was higher in male patients. 52.9% (9/17) of all patients had a history of psychiatric disorders, among which schizophrenia (*n* = 3), mood disorder (*n* = 4) and personality disorder (*n* = 2). All 4 patients with mood disorder had major depression and 2 patients with personality disorder had borderline personality disorder. Among the 8 patients with deep injuries, 6 had a history of psychiatric disorders, whereas among the 9 superficially injured patients, only 3 had a previous psychiatric diagnosis. When it comes to the long-term outcomes, 4 patients showed functional deficit in long-term follow-up period and all of these patients had nerve injuries including injuries of the median and the ulnar nerve. (Patient no. 1, 2, 3 and 5) 3 of the patients could not be evaluated properly due to follow-up loss.

**Table 1:** Summary of epidemiological and clinical characteristics of 17 cases

No	Sex	Side	Injured structure	Distance from wrist crease (cm)	Psychiatric disorder	Alcohol/ drug consumption	Postoperative Long-term disabilities
1	M	L	PL, median n.	4.5	–	+	+
2	M	L	APL, EPL / PL, 3rd to 5th FDS, median n.	5.8 / 3.7	+	–	+
3	M	L	FCU, Ulnar n., Ulnar a.	1.2	+	–	+
4	M	L	FCR, PL, 2nd & 3rd FDS, median n.	2.9	+	–	–
5	M	L	PL, median n.	5.7	+	–	+
6	M	L	FCR, radial a.	2.6	+	–	(Follow up loss)
7	M	R	4th FDP & FDS	2.8	+	–	–
8	M	R	FCR, FPL	4.2	–	–	–
9	F	R/L	Both PL	2.4 / 3.4	+	–	–
10	M	L	EPL	4.8	–	+	–
11	M	L	PL, 2nd to 5th FDS	3.1	–	+	(Follow up loss)
12	F	L	FCR	1.7	+	+	–
13	M	L	PL, FCR, FDS	2.8	–	–	–
14	F	R/L	Right APL, EPB / Left APL	4.7 / 3.2	–	–	–
15	M	L	PL, FCU, FCR	3.9	+	–	(Follow up loss)
16	M	R/L	PL	2.2 / 2.7	–	+	–
17	F	L	FCR, PL	3.7	–	–	–

(PL = palmaris longus tendon; FCR = flexor carpi radialis tendon; FCU = flexor carpi ulnaris tendon; FDS = flexor digitorum superficialis tendon; FDP = flexor digitorum profundus tendon; APL = abductor pollicis longus tendon; EPL = extensor pollicis longus tendon; FPL = flexor pollicis longus tendon; a. =artery; n. = nerve)

**Table 2:** Implements used in each case

Implement	Number of cases (17 cases)
Knife	9
Glass	4
Cutter	2
Scissor	1
Razor	1

## Discussion

Suicide is a global public health problem that impacts individuals and society. Suicide rates have increased substantially over the past two decades. Suicide in Korea is the tenth highest in the world according to the World Health Organization, making it the fourth leading cause of death [3]. Due to increasing cases of self-inflicted wrist cutting and its low mortality rate, initial evaluation when facing this injury is the most important aspect to prevent long-term functional impairment. Thus, we suggest appropriate evaluation and aim to share the perspectives of a hand surgeon.

### Demographics and psychiatric disorders of patients

In this study, there were gender differences in self-inflicted wrist cutting (4 female and 13 male patients), as opposed to other studies which showed a higher proportion of women with self-cutting injuries [7, 10]. This suggests that deep injuries involving deep flexor tendons, artery and nerve are more likely to occur in male patients. Male wrist-cutting patients showed more extensive injuries and all patients who had deep structural injuries were also male. 6 of the 8 deeply injured patients were previously diagnosed with psychiatric disorders. Furthermore, as patients with psychiatric disorder have a higher rate of recurrent suicide attempts [11], multidisciplinary approach together with the Psychiatric Department is essential in order to effectively treat these patients. Schizophrenic patients are more likely to get devastating injuries and other psychiatric disorders including depression and borderline personality disorder have a higher risk of attempted suicide [12]. Even though some patients have not been previously diagnosed with psychiatric disorders, it is likely that they have an underlying mental problem [13]. A thorough clinical assessment of the patient’s wound and situation is necessary to distinguish deliberate self-harm from unintentional or accidental injuries [14]. In this study, two patients with the deepest injuries (Patient no. 2, 3) were schizophrenic patients. Therefore, in case of male patient who have attempted suicide by wrist cutting and who have been diagnosed with a psychiatric disorder such as schizophrenia, the high possibility of deeper injury should be considered.

### Characteristics of injuries

**Fig. 1:**
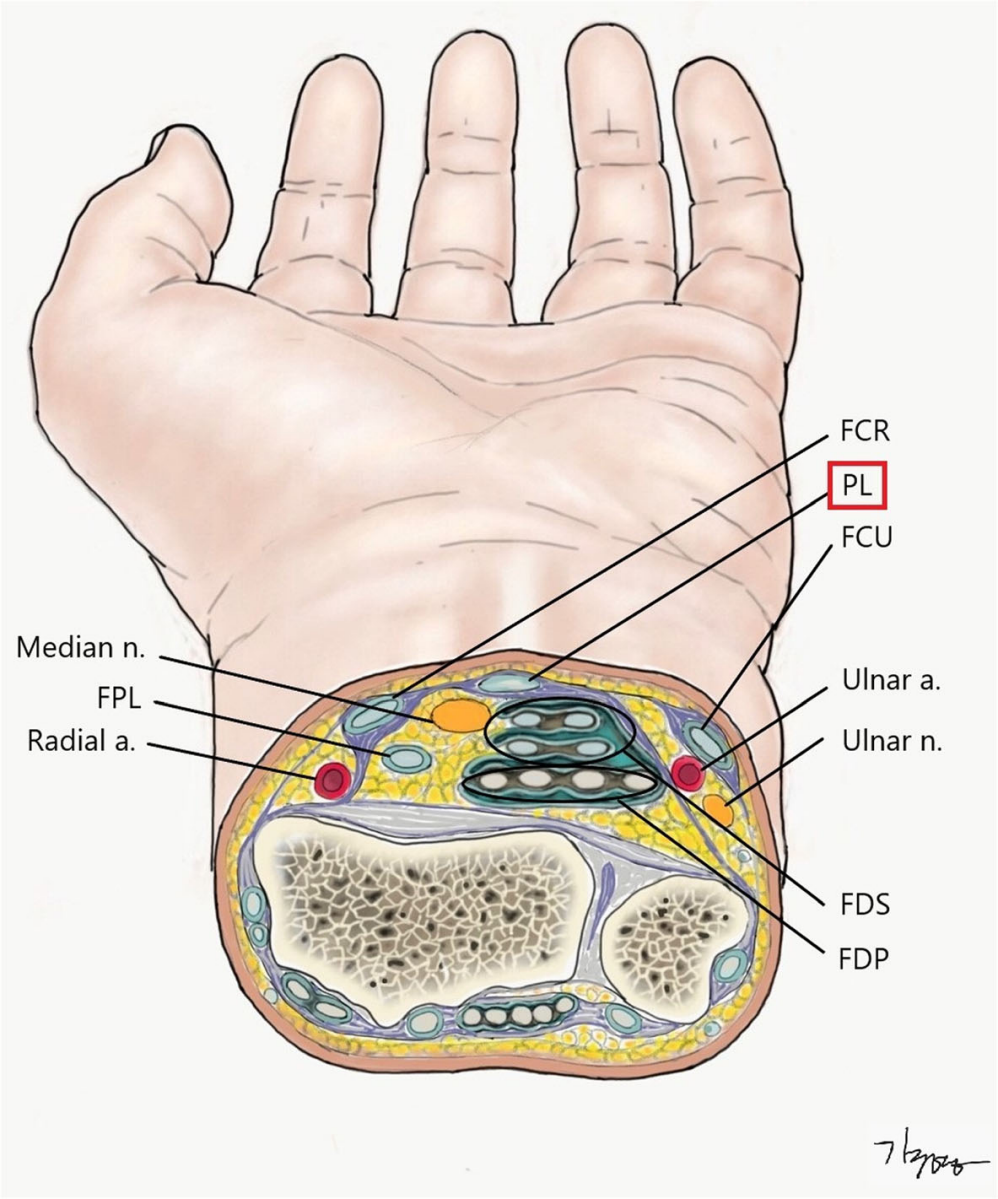
Axial anatomy of left wrist (of the most commonly injured level). (PL = palmaris longus tendon; FCR = flexor carpi radialis tendon; FCU = flexor carpi ulnaris tendon; FDS = flexor digitorum superficialis tendon; EPL = extensor pollicis longus tendon; FPL = flexor pollicis longus tendon; a. =artery; n. = nerve)

Ironically, this unpredictable trauma can be one of the most predictable injuries to a hand surgeon in three ways. First of all, as there are more right-handed people who hold implements with their right hands, there is higher probability of injury to the left wrist [7], as confirmed in our study, which showed injuries to the left wrist in 70.6% of cases (12/17). Secondly, almost all patients have flexor side injuries (16 patients with injuries of the flexor side and one with injury of the radial side). Wrist flexor tendons were the most frequently injured anatomical structures because they are located close to the skin surface and therefore more likely injured. Interestingly, the distance from the wrist crease to the injury site was mostly limited to 5 cm from the wrist crease. As can be seen through the results of this study, 90.5% (19/21) of injuries were limited in this area. The author drew an axial anatomy of the left wrist focusing on the most common injury site (Fig. 1) which could be helpful when it comes to understanding the anatomical relationship of important structures for those who are not familiar with the anatomy of this zone. Arterial bleeding from both the radial and the ulnar side on the wrist indicates the high probability of structural injuries in intentional self-cutting [15]. In case of injury of the ulnar artery, concomitant injuries of the flexor carpi ulnaris and/or of the ulnar nerve are likely to be observed (Patient no.5 in our series). Similarly, injury of the radial artery is often accompanied by an associated injury of the flexor carpi radialis tendon (Patient no.3 in our series). Thus, we should keep in mind the possibility of accompanying structural injuries if arterial bleeding is suspected in the patient.

### Consideration of implements and mechanism of injury

When we consider the type of implements used and mechanism of injuries, they can be classified into cutting wounds and stabbing wounds [9]. From an anatomical perspective, cutting wounds can be considered as horizontal injuries and stabbing wounds as vertical injuries. In terms of the implements used, Knife was the most common tool for suicide attempts, followed by glass. In cases of injuries caused by cutter and razor, they were all horizontal injuries, whereas in case of injuries caused by glass or scissors, vertical injuries were observed. Injuries by knife could be of both injury types, but cutting wounds were more common (8 cases of horizontal injuries and 1 case of vertical injury). Especially, if the patient has vertical injury on their wrists, more attention should be paid to the motor and sensory evaluation. In case of Patient No.7, the injury on the right wrist (glass) resulted in a wound of 1.5 cm in length. At initial examination, no specific functional deficits were detected and the patient was treated with primary wound closure in the emergency department. At further examination in our outpatient clinic, indication to surgical exploration was given: intraoperatively injuries to the 4th flexor digitorum superficialis and profundus tendons were detected and repaired. Since this kind of injury is often inconspicuous, proper evaluation is necessary depending on the mechanism of injury and implements used. Especially, when vertical injury is suspected, it is important to check the injured area through proper exploration.

### Appropriate initial evaluation of wrist cut injury

As mentioned above, initial evaluation and proper treatment are the most important in the prevention of long-term functional impairment. Accordingly, it would be helpful to undertake a proper evaluation in order to understand the axial anatomy schematically at the level where wrist cutting injury occurs most commonly. From the point of view of an initial examiner, not a hand surgeon, it is one of the best ways to estimate the possibility of structural injury by using the palmaris longus tendon, which is the most prominent structure on the flexor side, as an anatomical landmark (Fig. 1). The median nerve is located in the relatively shallow depth directly below the palmaris longus tendon. From a total of 17 patients in this study, there were 4 patients who inflicted damage on the median nerve, which was most commonly damaged nerve, and injury of the palmaris longus tendon was accompanied in all these cases. Of the 4 cases resulting in long-term disabilities, 3 of those cases were patients with median nerve damage which was the most likely structural injury to cause motor or sensory impairment (Patient No. 1, 2, 5). Regarding the palmaris longus tendon as the central structure, the flexor side of the wrist can be divided into the radial and the ulnar sides. FCR (flexor carpi radialis tendon) and FCU (flexor carpi ulnaris tendon) can be regarded as tendon group of superficial layer and it is relatively simple to detect the presence of injuries. Considering FCR and FCU as landmarks respectively, ulnar artery and nerve underneath FCU and radial nerve underneath FCR are important structures in the deep layer. When injury to FCR or FCU is detected, accompanying injuries in the deep layer should be considered. Furthermore, as mentioned above in case of arterial bleeding, we should also consider injuries of the adjacent structure such as the accompanying damage to both ulnar artery and nerve (Patient no.3).

This study has several limitations. First, there is a possibility of selection bias because this was a retrospective study and only patients who underwent operation at the Plastic and Reconstructive Department were included. Second, a sample size was small for statistical analysis because this was a preliminary study before we started prospective cohort study. In the future, a prospective studies using larger number of patients will be required. Despite these limitations, this study is meaningful in that it allows all stakeholders to understand the clinical characteristics of self wrist-cutting injuries and evaluate properly. A further prospective study will analyze the results of long-term follow-up and rehabilitation program of these patients, which could be more helpful for those who treat these patients primarily.

## Conclusions

In this study, we investigated 17 patients who had structural injury due to self-inflicted wrist-cutting as a pilot study. This demonstrated a different tendency in comparison with those with deep injuries. Male patients with a psychiatric disorder had a higher risk of more extensive wrist lacerations. We could also conclude that self-cutting injuries are highly predictable because most of these injuries occur on the flexor side of left wrist and are limited to a distance of 5 cm from the wrist crease. In terms of implements used when inflicted injury, we can predict the type of damage to some degree depending on the type of implement used. In view of these characteristics, more appropriate evaluation can be possible in the emergency room. In conclusion, through sharing our perspectives as hand surgeons, we can aid those who face these patients primarily, allowing them to cope more effectively for better long-term results.

## Data Availability

Data has been anonymized and is kept with the authors. It is available upon request.

## Abbreviations

FCR: flexor carpi radialis tendon
FCU: flexor carpi ulnaris tendon
